# Thermal Shock and Ciprofloxacin Act Orthogonally on *Pseudomonas aeruginosa* Biofilms

**DOI:** 10.3390/antibiotics10081017

**Published:** 2021-08-21

**Authors:** Haydar Aljaafari, Yuejia Gu, Hannah Chicchelly, Eric Nuxoll

**Affiliations:** 1 Department of Chemical and Biochemical Engineering, 4133 Seamans Center for the Engineering Arts and Sciences, University of Iowa, Iowa City, IA 52242, USA; haydar-aljaafari@uiowa.edu (H.A.); yuejia-gu@uiowa.edu (Y.G.); hannah.chicchelly@gmail.com (H.C.); 2 Department of Chemical Engineering, University of Technology, Baghdad 10066, Iraq

**Keywords:** biofilms, prosthesis-related infections, heat shock, ciprofloxacin, antibacterial agents

## Abstract

Bacterial biofilm infections are a major liability of medical implants, due to their resistance to both antibiotics and host immune response. Thermal shock can kill established biofilms, and some evidence suggests antibiotics may enhance this efficacy, despite having an insufficient effect themselves. The nature of this interaction is unclear, however, complicating efforts to integrate thermal shock into implant infection treatment. This study aimed to determine whether these treatments were truly synergistic or simply orthogonal (i.e., independent). *Pseudomonas aeruginosa* biofilms of different architectures and stationary-phase population density were subjected to various thermal shocks, antibiotic exposures, or combinations thereof, and examined either immediately after treatment or after subsequent reincubation. Population decreases from the combination treatment matched the product of the decreases of individual treatments, indicating their orthogonality. However, reincubation showed binary behavior, where biofilms with an immediate population decrease beyond a critical factor (~10^4^) died off completely during reincubation, while biofilms with a smaller immediate decrease regrew. This critical factor was independent of the initial population density and the combination of treatments that achieved the immediate decrease. While antibiotics do not appear to enhance thermal shock directly, their contribution to achieving a critical population decrease for biofilm elimination can make the treatments appear strongly synergistic, strongly decreasing the intensity of thermal shock needed.

## 1. Introduction

More than 750,000 knee replacement and 500,000 hip replacement surgeries are performed each year in the United States [1], and these numbers are expected to increase exponentially in the next decade [2]. In total, 1% to 4% of the knee replacement and 1% to 2% of the hip replacement procedures are followed by incidences of periprosthetic joint infection [3,4,5]. The pathogens in these infections typically organize themselves in a densely populated thin layer of polysaccharides, proteins, and DNA called a biofilm, in which they exhibit a phenotype 20–100 times more resistant to antibiotics and host immune response than their planktonic phenotype [6,7,8,9,10]. Thus, the current standard of care is high doses of antibiotics and surgical explantation of the implant with its surrounding infected tissue, followed eventually by implantation of a replacement device [11,12,13]. Though this is successful in over 90% of cases [14,15], the new implant has a higher risk of infection than the original one [16]. These multiple invasive procedures expose the patient to physical risk and low quality of life, in addition to significant financial costs [11,17]. The incidence of infection has persisted despite decades of effort to create surfaces that prevent biofilm formation [18,19,20,21,22,23,24] and to develop methods to eradicate established biofilms [25,26,27,28,29,30,31,32], none of which have progressed to clinical implementation.

Thermal shock has been demonstrated as a means of deactivating established biofilms but may also damage adjacent tissue [33,34]. Recent studies have suggested a synergism between antibiotics and thermal shock, with the combined treatment decreasing biofilm population density more than either treatment alone, or even by the product of their individual effects [35,36,37,38]. The nature of this interaction is poorly understood. One hypothesis is that biofilms may have a critical population density below which they become non-viable, and that any combination of approaches that drops the population below that level will result in complete elimination of the biofilm, even if the individual approaches are not nearly so effective. This study investigated this hypothesis in *Pseudomonas aeruginosa* biofilms. Using biofilms of significantly different architecture and initial population density, it demonstrates not a critical population level but rather a critical population decrease, beyond which the population proceeds to zero.

Two different protocols were used to culture *P. aeruginosa* biofilms with significantly different population density and architecture: Shaker table (ST) and drip flow reactor (DFR). Biofilms of each type were subjected to thermal shocks ranging from 50 °C for 1 min to 70 °C for 30 min, with or without 4 h of prior exposure to ciprofloxacin (CP) concentrations ranging from 0.25 to 64 μg/mL. Shocked biofilms were either immediately enumerated or reincubated for 1 or 2 days before enumeration. Biofilms of each type were similarly exposed to antibiotics and re-incubation without thermal shock.

## 2. Results

### 2.1. Population Density, Architecture, and Thermal Susceptibility

**Figure 1:**
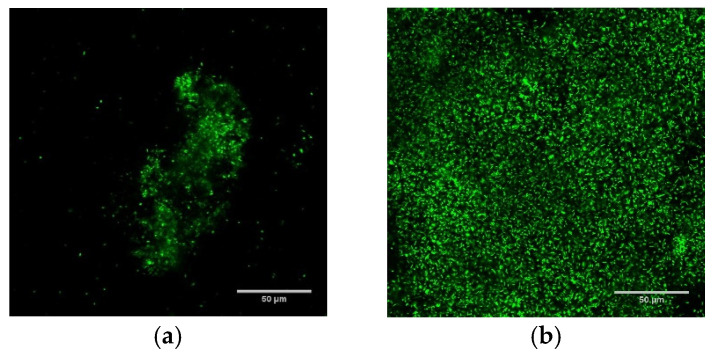

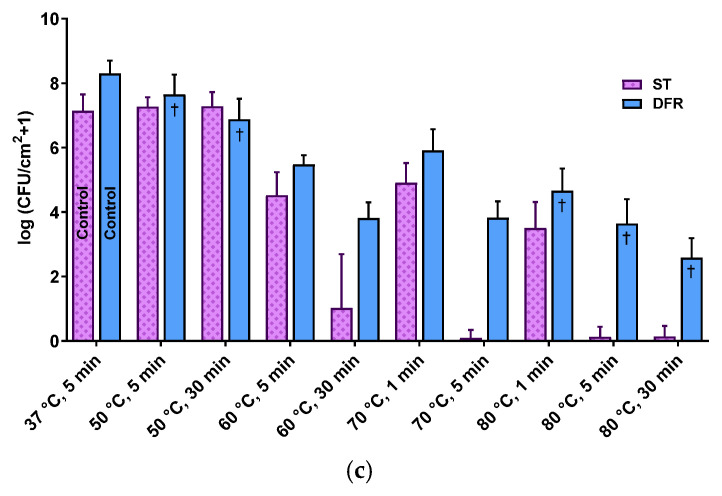
Culture protocols produce biofilm with starkly different population density, architecture, and thermal susceptibility. (**a**,**b**) are overhead views of confocal fluorescent images of shaker table (**a**) and drip flow reactor (**b**) biofilms. (**c**) Effect of thermal shock on population density of *P. aeruginosa* ST and DFR biofilms. † from [35].

Biofilms grown using DFR and ST protocols had markedly different population densities (as measured in colony forming units (CFU) per cm^2^), spanning from sparsely populated (10^7.13±0.58^ CFU/cm^2^) biofilms with individual micron-scale features ~50 μm in height (ST) to densely populated (10^8.3±0.4^ CFU/cm^2^) carpets over 100 μm thick (DFR). Figure 1 demonstrates these architectural differences with confocal fluorescent microscopy images of ST (panel a) and DFR (panel b) biofilms, as well as comparing their population densities (panel c).

The thermal susceptibility of the biofilms was similarly different at modest temperatures (50 °C), with DFR biofilms decreasing by up to 1.5 orders of magnitude while ST biofilms showed no effect. At high temperatures (80 °C), however, the thermal susceptibility of the different biofilms converges, with both biofilm types decreasing by 3.5 orders of magnitude after 1 min of exposure. For longer exposures, ST biofilm populations appear to drop off completely.

### 2.2. Re-Incubation

**Figure 2:**
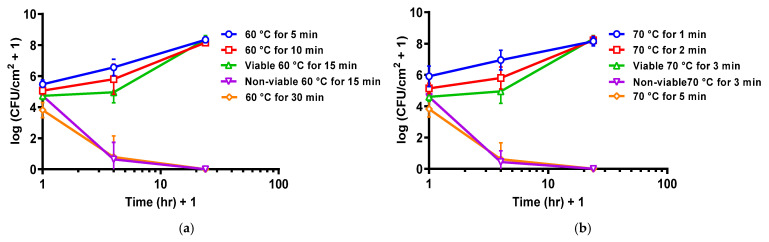
Critical population density for DFR biofilm reincubation. (**a**) Population density for DFR biofilm reincubation after thermal shock at 60 °C. (**b**) Population density for DFR biofilm reincubation after thermal shock at 70 °C. Error bars indicate standard deviation for at least six slides from three different dishes.

Re-incubation of thermally shocked DFR biofilms showed two opposing trends. After shocks sufficient to drop the population density below 10^4.5^ CFU/cm^2^, the population density continued to decrease during re-incubation, dying off completely within a few hours. After milder thermal shocks, however, the biofilms regrew during reincubation, achieving their previous stationary-phase population density within a day. This is shown graphically in Figure 2 for eight thermal shock conditions, two of which typically dropped DFR biofilms to ~10^4.5^ CFU/cm^2^. At those two conditions, both trends can be observed, with 60% (6 of 10) of the biofilms shocked at 60 °C for 15 min dying off while the remainder grew back (Figure 2a), and 66% (8 of 12) of the biofilms shocked at 70 °C for 3 min dying off while the remainder grew back (Figure 2b).

### 2.3. Antibiotic Exposure

**Figure 3:**
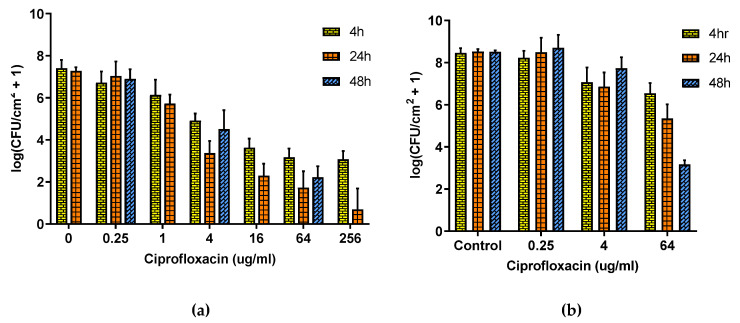
Effect of Ciprofloxacin on ST and DFR biofilms. (**a**) Effect of Ciprofloxacin on ST biofilm. (**b**) Effect of Ciprofloxacin on DFR biofilm. Error bars indicate standard deviation for at least six slides from three different dishes.

Figure 3 shows that the relationship between the *P. aeruginosa* population density and CP concentration follows a power law for both DFR and ST biofilms, albeit shifted, with the same dosage having less efficacy on DFR biofilms than on ST ones. Panel (a) shows that for ST biofilms, four hours of exposure failed to decrease the population density by four orders of magnitude even at concentrations over 60 times higher than physiological dosing 4 μg/mL [39]. Increasing the exposure time to 24 h increased efficacy but still did not eliminate the biofilm, and with further exposure (48 h) the population density actually recovered rather than further decreased. The effect of CP is much smaller on DFR biofilms (Panel b) with only modest decreases in population density even at grossly toxic concentration (64 μg/mL) and long exposure times, though under these circumstances, the population density is at least still trending downward with exposure time.

### 2.4. Combined Antibiotic and Thermal Exposure of Shaker Table (ST) Biofilms

**Figure 4:**
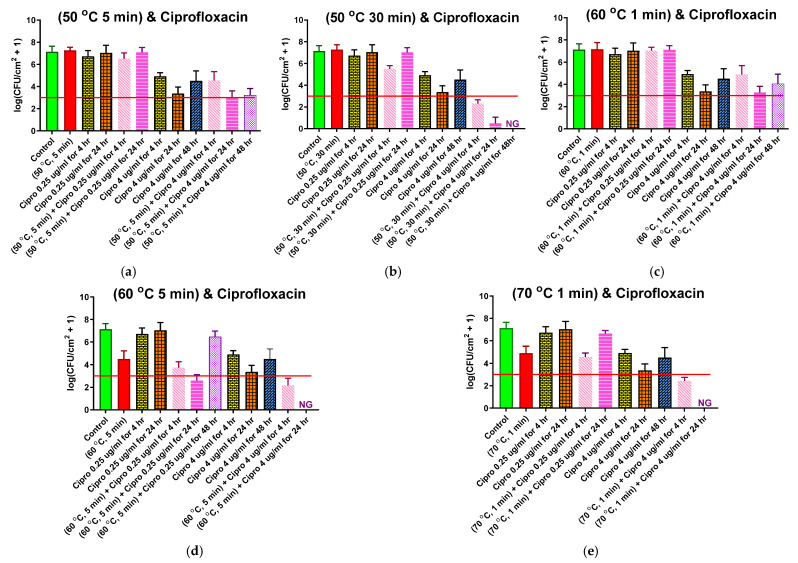
Combined ciprofloxacin and thermal shock effect on the *P. aeruginosa* shaker table biofilm population. Each panel shows results for the thermal shock and antibiotic exposure indicated. Red horizontal lines show the critical population density below which thermal shocked bacterial biofilms are not viable. Error bars indicate standard deviation for at least six slides from three different dishes.

Five different thermal shock protocols (50 °C for 5 or 30 min, 60 °C for 1 or 5 min, 70 °C for 1 min) were applied to ST biofilms after 4 h of CP (0.25 or 4.0 μg/mL) exposure at 37 °C. For each protocol and CP concentration, three reincubation conditions were investigated: 0, 24, or 48 h in fresh media at the designated CP concentration. The results for each protocol are shown in their own panel of Figure 4, alongside controls with no thermal shock. These controls include: No treatment (first, or leftmost, bar of each panel), with an average pre-treatment population density of 10^7.13^ CFU/cm^2^; 0.25 μg/mL CP exposure for 4 or 24 h (3rd and 4th bars, respectively), showing that this exposure by itself had no significant effect on population density; and 4 μg/mL CP exposure for 4, 24, or 48 h (7th, 8th, and 9th bars, respectively), showing that, by itself, this maximum physiological dose of ciprofloxacin only reduced the population density to 10^4.92 ±0.33^ CFU/cm^2^ after 4 h of exposure and 10^3.37±0.58^ after 24 h of exposure, recovering back to 10^4.51±0.9^ after 48 h of exposure.

The second bar of each chart shows the effect of the thermal shock by itself (i.e., no antibiotic exposure), with only the shocks at 60 °C for 5 min (Figure 4d) or 70 °C for 1 min (Figure 4e) showing any significant effect. Combining thermal shock with exposure to 0.25 μg/mL CP, thermal shocks that had no effect by themselves also had no effect when added with 0.25 μg/mL antibiotic. There is a significant effect for the 50 °C for 30 min (Figure 4b) shock after 4 h of antibiotic exposure, but this disappeared with 24 h of exposure and may be an anomaly. For thermal shocks that did have a significant effect by themselves, 4 h of exposure to 0.25 μg/mL CP may have increased the effect, albeit not significantly. Further exposure for 24 h has conflicting results, with the enhancement becoming significant with a 60 °C shock for 5 min (Figure 4d), while allowing regrowth after 70 °C shocks for 1 min (Figure 4e). No consistent synergism (or antagonism) is seen.

**Figure 5:**
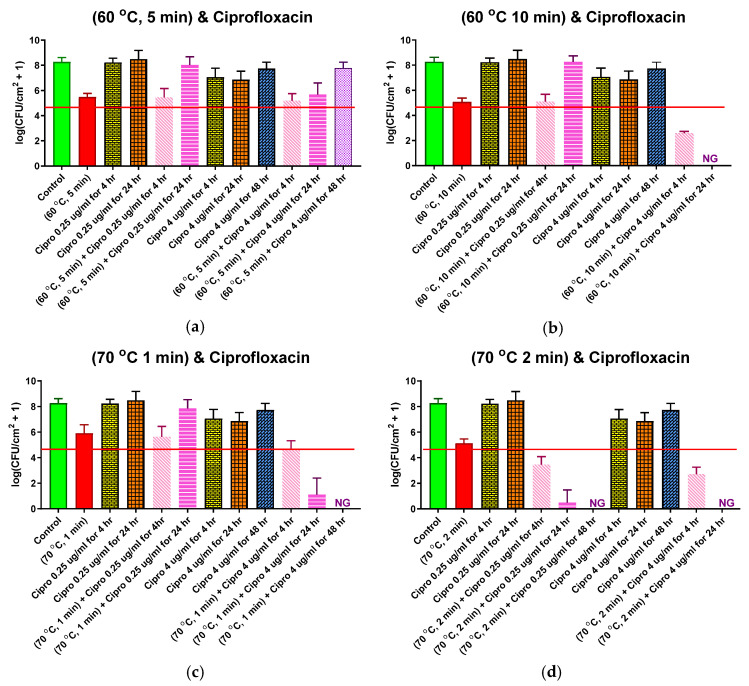
Combined ciprofloxacin and thermal shock effect on the *P. aeruginosa* drip flow reactor biofilm population. Each panel shows results for the thermal shock and antibiotic exposure indicated. Red horizontal lines show the critical population density below which thermal shocked bacterial biofilms are not viable. Error bars indicate standard deviation for at least six slides from three different dishes.

Combining thermal shock with exposure to 4.0 μg/mL CP, shocks at 50 °C for 5 min (Figure 4a) and 60 °C for 1 min (Figure 4c) appear to have no effect; the results are essentially the same with or without the shock. Since these shocks also had no effect by themselves, one can state that the effects of the two treatments are additive, with the log reduction of the combined treatment matching the sum of the log reductions for each treatment by itself. Similarly, for more aggressive shocks (60 °C for 5 min, or 70 °C for 1 min), both the thermal shock and the 4 h exposure to 4 μg/mL CP have an effect by themselves, and the log reduction in population density for the combined treatment roughly matches the sum of the log reductions for each individual treatment. Only for the intermediate shock at 50 °C for 30 min (Figure 5b) does the combined treatment prompt a log reduction larger than the sum of the individual treatments, suggesting a synergism between the treatments.

Additional exposure to 4 μg/mL CP after these more aggressive thermal shocks has a dramatically different result, however. After 24 h of exposure, the biofilms shocked at 60 °C for 5 min or 70 °C for 1 min have no detectable CFU, see Figure 4d,e respectively. After 48 h, the biofilm shocked at 50 °C for 30 min (Figure 4b) also has no detectable CFU. These treatments appear highly synergistic.

### 2.5. Combined Antibiotic and Thermal Exposure of Drip Flow Reactor (DFR) Biofilms

Four different thermal shock protocols (60 °C for 5 or 10 min, 70 °C for 1 or 2 min) were applied to DFR biofilms in combination with subsequent CP (0.25 or 4.0 μg/mL) exposure at 37 °C for 4, 24, or 48 h. These protocols were chosen because they each cause a large population density reduction in DFR biofilms but not so large that the biofilm eventually dies off, as seen in Figure 2. The results for each protocol are shown in their own panel of Figure 5, alongside controls with no thermal shock. These controls include: No treatment (first, or leftmost, bar of each panel), with an average pre-treatment population density of 10^8.3^ CFU/cm^2^; 0.25 μg/mL CP exposure for 4 or 24 h (3rd and 4th bars, respectively), showing that this exposure by itself had no significant effect on population density; and 4 μg/mL CP exposure for 4, 24, or 48 h (7th, 8th, and 9th bars, respectively), showing that, by itself, this maximum physiological dose of CP only reduced the population density by an average log reduction of about 1.5 after 4 or 24 h of exposure, and that biofilms actually recovered to approximately their pre-treatment population density within 48 h, despite continuous antibiotics exposure. 

The second bar of each chart shows the effect of the thermal shock by itself (i.e., no antibiotic exposure), with log reductions of 2.3–3.4. Adding four hours of exposure to 0.25 μg/mL CP, the thermal shock had virtually no additional effect except at the highest temperature for its longest shock (70 °C for 2 min), where an additional log reduction of 1.7, to 10^3.5^ CFU/mL, was observed (Figure 5d). Increasing the antibiotic exposure to 24 h simply allowed the biofilm to grow back to its pre-treatment population density, except in the latter case, where the bacteria continued to die off, with no detectable CFU after 48 h. 

Adding four hours of exposure to 4.0 μg/mL CP after the thermal shock, however, reduced the population density beyond the thermal shock alone in each case. For the shorter shocks at each temperature (Figure 5a,c), the log reductions of the combined treatments were slightly less than the sum of the log reductions of each treatment alone, see Figure 5b,d. The longer shocks, which had larger log reductions by themselves, also had larger log reductions from the combined treatment, even larger than the sum of the individual treatments. With longer antibiotic exposure, the biofilm population plummeted for most cases, with no detectable CFU after 24 h for the longer shocks at each temperature, and no detectable CFU after 48 h for the 1 min shock at 70 °C. Only for the 5 min shock at 60 C°, which had the highest population density after 4 h of 4 μg/mL CP exposure, did the biofilm regrow with continued antibiotic exposure, eventually (48 h) approaching its pre-treatment population density. Like the ST biofilms, these results suggest significant synergism between thermal shock and antibiotic exposure.

## 3. Discussion

Biofilm infections are particularly problematic because they cannot be eliminated by our primary in vivo treatment against bacteria, antibiotics. Ciprofloxacin’s ability to eliminate this strain of bacteria in the planktonic phenotype has been previously reported [35]. In the biofilm phenotype, however, dramatically higher drug concentrations are needed for comparable population reduction. While this reduction follows a power-law relationship with concentration, it levels out without elimination of the biofilm, as reported previously for MBEC assay biofilms [35] and indicated here in Figure 3 for ST biofilms, where at grossly toxic antibiotic concentrations, the population density appears to asymptote at ~10^3^ CFU/cm^2^ after 4 h. The population density decreases further over the next 20 h but recovers again over the following day. For DFR biofilms, the decrease is irrelevant even at grossly toxic concentrations. Other approaches are clearly needed for biofilm infection mitigation.

Thermal shock is known to kill biofilms, but implementation in vivo requires minimizing the severity of the shock in order to minimize the accompanying damage to the adjacent tissue. Previous reports have indicated that antibiotics increase the efficacy of thermal shock, mitigating biofilms with less aggressive thermal treatment [35]. In some cases, the two approaches appear to be synergistic, with the reduction from the combined treatment exceeding the sum of the reductions from the individual treatments. The nature of this synergism was unknown, so this project aimed to investigate it, specifically scrutinizing the effect of re-incubation, with or without antibiotics, for biofilms spanning a wide range of architecture and population density.

As discussed earlier, the ST and DFR growth protocols involve starkly different growing conditions, which provide biofilms with dramatically different population density, structure, and maturity, as illustrated in Figure 1. These differences also prompt different thermal susceptibilities, with DFR biofilms strongly impacted by 50 °C exposure while ST biofilms are unaffected by it. At higher temperatures, however, their susceptibility (as measured by immediate population decrease) converges.

Immediate population decrease does not appear to completely quantify thermal susceptibility, though. After the thermal shock is removed and incubation conditions are restored, some DFR biofilms will recover to their pre-treatment population density, while others will continue to die off until no CFU are detectable. Figure 2 shows that this behavior can be predicted from the population density immediately after the thermal shock. DFR biofilms with a population density above ~10^4.5^ CFU/cm^2^ grow back, while biofilms with a population density below that value die off, regardless of the temperature of the shock. Similar behavior was previously reported for ST biofilms, but in that case, the critical population density was ~10^3^ CFU/cm^2^ [40]. Notably, this difference in critical population density (by a factor of ~10^1.5^) is the same as the difference in the initial population densities of the two biofilms. In both cases, when the population density dropped by over four orders of magnitude, the biofilm died off, while smaller population density reductions resulted in the biofilm growing back completely, no matter what temperature and exposure time were used to achieve the reduction. These results suggest that mitigating biofilms is not based on driving its population density below a particular critical quorum, but rather achieving a particular population density reduction. This is encouraging since, at higher temperatures, this reduction appears to be the same regardless of the type of biofilm or its initial population density, as shown in Figure 1.

Figure 4 and Figure 5 suggest that this re-incubation behavior is responsible for the perceived synergism between thermal shock and antibiotics. Each panel in Figure 5 includes a horizontal line at the critical population density identified in Figure 2. Similarly, each panel in Figure 4 includes a horizontal line at the critical population density previously reported for ST biofilms [40]. Looking at combination treatments that resulted in populations above the critical value after 4 h, the decrease in population density for the combined thermal shock + antibiotics treatment is not significantly different than the sum of the decreases by thermal shock alone and antibiotics alone. The only exception to this in either figure is for ST biofilms exposed to 0.25 μg/mL ciprofloxacin and shocked at 50 °C for 30 min. In every other case, the treatments appear to be simply additive, indicating that their mechanisms are orthogonal and non-overlapping but not synergistic. Even for the 50 °C/30 min/0.25 μg/mL case, the enhanced decrease disappears within 24 h. 

On the other hand, when the population reduction of the combined thermal shock + antibiotic treatment brings the population density below the critical value, this reduction is significantly larger than the sum of the reductions of its individual components, and this difference becomes pronounced at longer times as the population of CFU drops to zero. This is demonstrated five times in Figure 4 (50 °C for 30 min with 4 μg/mL; 60 °C for 5 min with 0.25 μg/mL) and Figure 5 (60 °C for 10 min with 4 μg/mL; 70 °C for 1 min with 4 μg/mL; 70 °C for 2 min with 0.25 μg/mL) with a wide range of temperature/time/antibiotic combinations, showing that this is independent of the original architecture of the biofilm and the manner in which the critical decrease is achieved. There are three instances where the reduction at 4 h is beyond the critical reduction but not significantly different than the sum of component reductions (ST biofilms shocked at 60 °C for 5 min or 70 °C for 1 min and DFR biofilms shocked at 70 °C for 2 min, all exposed to 4 μg/mL ciprofloxacin), but in all three cases, the biofilms proceed to die off within 24 h, resulting in a population reduction that is again much larger than the sum of the heat-shock only and antibiotics-only reductions. While neither treatment by itself achieved the critical population drop, the combination of the treatments did, prompting further population decrease, which makes the treatment appear synergistic, even though there is no indication that the treatments actually interact in any way.

In summary, previous studies have suggested a synergistic link between antibiotic exposure and thermal shock in the eradication of bacterial biofilms, i.e., that one treatment enhances the efficacy of the other in some way, resulting in a population reduction larger than predicted from simply adding the effects of the treatments by themselves. This study investigated that link, growing *P aeruginosa* biofilms of two disparate population densities and architectures and combining a variety of different thermal shocks with different concentrations of ciprofloxacin. When the sum of the log population decrease for thermal shock alone and for antibiotics alone was less than four orders of magnitude, the population decrease when combining the two treatments was not significantly different than the sum from the individual treatments, indicating no synergism, just orthogonal mechanisms. However, when the sum from the individual treatments was more than four orders of magnitude, the decrease from the combined treatment was even larger, eventually eliminating the biofilm altogether. While this gives the appearance of synergistic interaction in just those instances, the same eventual elimination is observed even without antibiotic exposure when the initial population decrease exceeds four orders of magnitude from thermal shock alone. It appears likely that thermal shock and antibiotics act with strictly orthogonal mechanisms on *P aeruginosa* biofilms, but both contribute to a separate, general, critical decrease phenomenon.

## 4. Materials and Methods

### 4.1. Streak and Inoculum

*Pseudomonas aeruginosa* PAO1 (15692, American Type Culture Collection, Manassas, VA, USA) was streaked on an agar plate (Difco Nutrient Agar, Sparks, MD, USA) and incubated inverted for 24 h at 37 °C. Two colonies from the streaked plate were harvested and moved into 5 mL (30 g/L) Tryptic Soy Broth (TSB, Becton, Dickinson and Company, Franklin Lakes, NJ, USA) using a sterile inoculating loop. Inoculated TSB was incubated for 24 h at 37 °C to form an inoculum with an average of ~10^9^ colony forming units (CFU) per mL.

### 4.2. Biofilm Culture

#### 4.2.1. Shaker Table Biofilm

In total, 0.333 mL of inoculum and 5 mL of 30 g/L TSB were added to each well of 4-well dishes (Thermo Fisher Scientific, Waltham, MA, USA). In each well, a microscope slide fully frosted on one side, 75 mm × 25 mm × 1 mm (Leica Biosystems, Buffalo Grove, IL, USA) was immersed, and then the dish was sealed with parafilm. Dishes were placed on an orbital shaker table (VWR 1000, 15 mm orbit, Radnor, PA, USA) set at 160 rpm, and placed inside an incubator at 37 °C for 96 h to culture mature ST biofilms.

#### 4.2.2. Drip Flow Reactor (DFR) Biofilm

DFR biofilm culture includes two distinct modes, batch and continuous. A frosted microscope slide was immersed in 15 mL (30 g/L) TSB in each well of a 4-well reactor (Biosurface Technologies Corporation, Bozeman, MT). In total, 1 mL of inoculum was added to each well, then the wells were sealed with their lids. During batch mode, the reactor was at rest horizontally inside a 37 °C incubator for 4 h. To begin continuous mode, the reactor was tilted by 10 degrees and a steady drip of TSB (3 g/L) was applied to the upper end of the nascent biofilm, flowing down the slide by gravity to a drain hose at the lower end. The continuous mode ran for 20 h inside the incubator at 37 °C, with a drip flowrate of 1.25 L/day per well.

### 4.3. Thermal Shock

Mature ST or DFR biofilms were removed from their culture wells and rinsed in 5 mL of 3 g/L TSB for 1 min to remove planktonic bacteria, and then transferred to preheated 4-well dishes with 5 mL of 3 g/L TSB in each well. Biofilms were thermally shocked at (50, 60, or 70 °C) for (1, 2, 5, 10, or 30 min). Temperature was controlled by keeping the dishes in a water bath at the target temperature for 30 min prior to thermal shock and throughout the shock itself. Biofilms were transferred immediately after the thermal shock to new 4-well dishes with 5 mL of 3 g/L TSB per well at ambient temperature.

### 4.4. Re-Incubation

To investigate the viability of thermally shocked biofilms, they were re-incubated under conditions identical to their initial culturing. Thermally shocked DFR biofilms were placed in a fresh DFR inclined at 10°, in which the 3 g/L TSB drip. Biofilms were re-incubated for 4 or 24 h at 37 °C and the same flowrate of 1.25 L/day per well.

### 4.5. Antibiotic Exposure

In total, 5 mg/mL ciprofloxacin (CP) stock was prepared by dissolving ciprofloxacin hydrochloride (MP Biomedicals, Santa Ana, CA, USA) in de-ionized water. The stock was filtered with a 0.22 μm PES membrane sterile filter (Millex^®^GP filter unit) and stored at 2 °C. 

#### 4.5.1. Shaker Table Antibiotic Exposure

Mature ST biofilms were rinsed in 5 mL of TSB (3 g/L) for 1 min to remove planktonic bacteria, and then placed in a fresh 4-well dish, where each well contained 5 mL TSB (30 g/L) and CP (0.25, 1, 4, 16, 64, or 256 μg/mL). These concentrations were selected to observe antibiotic effects below, at, or above intravenous administration concentrations (4 μg/mL) on biofilm [39]. Biofilms were kept in these antibiotics challenge plates for 4, 24, or 48 h at 37 °C.

#### 4.5.2. Drip Flow Reactor Antibiotic Exposure

Mature DFR biofilms were placed in a fresh DFR inclined at 10°, in which the 3 g/L TSB drip also contained CP at 0.25, 4, or 64 μg/mL. The flowrate remained at 1.25 L/day per well for 4, 24, or 48 h at 37 °C.

### 4.6. Antibiotic and Thermal Exposure

To investigate the interaction of antibiotics and thermal shock on the reduction of biofilm population density, mature biofilms were exposed to antibiotics for four hours as described above. Following this exposure, biofilms were immediately transferred to preheated 4-well dishes with 5 mL of 3 g/L TSB and the same antibiotic concentration for thermal shock as described above. After the thermal shock, biofilms were either immediately enumerated, or reincubated in a new 4-well dish (ST) or new reactor (DFR) at 37 °C with the same antibiotics concentration for the remainder of a 24 or 48 h antibiotics exposure. 

### 4.7. Enumeration

Biofilms’ population density was determined via suspension and plating. Biofilms were transferred to a fresh 4-well dish containing 5 mL of 3 g/L TSB and mechanically disrupted and homogenized with a sonicator bath for 10 min at 45kHz (VWR Symphony, 9.5 L). The sonicated homogenous solutions were then serial diluted by 10 fold, and spot plated with 10 μL samples on nutrient agar plates. After 20 h of incubation, grown colonies were counted and the population density of colony forming units per square centimeter was calculated.

### 4.8. Confocal Microscopy

Confocal laser scanning microscopy was performed to evaluate ST and DFR biofilms’ architectural characteristics. Biofilms were exposed to fluorescent dyes from a LIVE/DEAD BacLight Bacterial Viability Kit (Invitrogen, Eugene, OR) for 20 min in low-light conditions to stain the biofilms prior to imaging with a non-inverted confocal microscope (Zeiss LSM 710, Oberkochen, Germany).

### 4.9. Statistical Analysis

Enumeration results were calculated on a log scale for statistical analysis. Significance was determined using two-tailed student-t tests with a 95% confidence interval. Variance for each trial arm is assumed to be uncorrelated, and differences in variance were reconciled per Cochran [41].

## 5. Conclusions

Reincubation studies on treated *P aeruginosa* biofilms indicate a critical population decrease beyond which the population will continue decreasing rather than recover. The factor by which thermal shock or ciprofloxacin reduce biofilm population density appear to be the same regardless of whether the other treatment factor is also applied, indicating that the reduction mechanisms are orthogonal (i.e., independent of each other) rather than synergistic. However, beyond the critical overall population decrease factor, the biofilm population continues to crash to zero, resulting in a post-treatment reduction far beyond the product of the individual treatments, making them appear synergistic even if their mechanisms do not actually overlap.

## Data Availability

The raw data collated and reported here is available in the NuxollResearchGroup DataVerse (URL and journal-specific dataset name to be added after manuscript acceptance).
